# *N*-Benzyl­carbamothioyl-2-chloro­benzamide

**DOI:** 10.1107/S1600536810023822

**Published:** 2010-06-26

**Authors:** Xi Zheng, Bo Li, Qiang Wang, Li Guo

**Affiliations:** a Key Laboratory of Drug Targeting and Drug Delivery Systems, Ministry of Education, West China School of Pharmacy, Sichuan University, Chengdu 610041, People’s Republic of China

## Abstract

In the title compound, C_15_H_13_ClN_2_OS, the dihedral angles between the sulfourea group and the benzene ring and the chloro­benzene ring are 35.8 (6) and 81.6 (6)° respectively. An intra­molecular N—H⋯O inter­action occurs. In the crystal, a combination of inter­molecular π–π stacking inter­actions [centroid–centroid distance = 4.0616 (16) Å] and N—H⋯S hydrogen bonds stabilizes the structure.

## Related literature

For general background to the chemistry and biological activity of thio­urea derivatives and their use, see: Jain & Rao (2003 ▶); Zeng *et al.* (2003 ▶); Xu *et al.* (2004 ▶); Zheng *et al.* (2004 ▶); D’hooghe *et al.* (2005 ▶); Saeed *et al.* (2008 ▶, 2009 ▶, 2010 ▶).
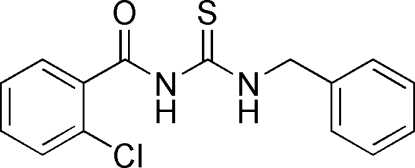


## Experimental

### 

#### Crystal data

C_15_H_13_ClN_2_OS
*M* *_r_* = 304.78
Triclinic, 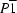

*a* = 7.347 (2) Å
*b* = 9.658 (3) Å
*c* = 11.003 (3) Å
α = 110.150 (5)°
β = 90.767 (3)°
γ = 104.058 (3)°
*V* = 707.0 (4) Å^3^
*Z* = 2
Mo *K*α radiation
μ = 0.41 mm^−1^
*T* = 153 K
0.40 × 0.30 × 0.30 mm

#### Data collection

Rigaku AFC10/Saturn724+ diffractometer
Absorption correction: multi-scan (*ABSCOR*; Higashi, 1995 ▶) *T*_min_ = 0.852, *T*_max_ = 0.886
5691 measured reflections
2481 independent reflections
2194 reflections with *I* > 2σ(*I*)
*R*_int_ = 0.017

#### Refinement

*R*[*F* ^2^ > 2σ(*F* ^2^)] = 0.029
*wR*(*F* ^2^) = 0.073
*S* = 1.01
2481 reflections
189 parameters
H atoms treated by a mixture of independent and constrained refinement
Δρ_max_ = 0.23 e Å^−3^
Δρ_min_ = −0.18 e Å^−3^

### 

Data collection: *CrystalClear* (Rigaku/MSC, 2005 ▶); cell refinement: *CrystalClear*; data reduction: *CrystalClear*; program(s) used to solve structure: *SHELXS97* (Sheldrick, 2008 ▶); program(s) used to refine structure: *SHELXL97* (Sheldrick, 2008 ▶); molecular graphics: *ORTEPIII* (Burnett & Johnson, 1996 ▶); software used to prepare material for publication: *PLATON* (Spek, 2009 ▶).

## Supplementary Material

Crystal structure: contains datablocks global, I. DOI: 10.1107/S1600536810023822/pk2248sup1.cif

Structure factors: contains datablocks I. DOI: 10.1107/S1600536810023822/pk2248Isup2.hkl

Additional supplementary materials:  crystallographic information; 3D view; checkCIF report

## Figures and Tables

**Table 1:** Hydrogen-bond geometry (Å, °)

*D*—H⋯*A*	*D*—H	H⋯*A*	*D*⋯*A*	*D*—H⋯*A*
N1—H1*N*⋯S1^i^	0.86 (2)	2.53 (2)	3.3698 (18)	166.2 (18)
N2—H2*N*⋯O1	0.82 (2)	2.01 (2)	2.669 (2)	137 (2)

Symmetry code: (i) 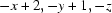.

**Fig. 1.**
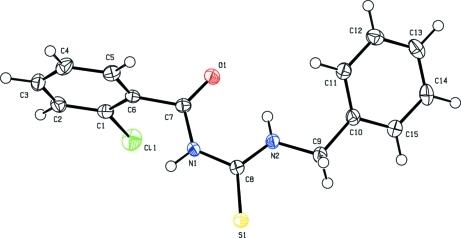
The molecular structure of the title compound, with displacement ellipsoids drawn at the 30% probability level.

## supplementary crystallographic information

### Comment

*N*-(benzylcarbamothioyl)-2-chlorobenzamide derivatives are of great importance owing to their wide-ranging biological properties (Zeng *et al.* (2003)). The title compound is one of the key intermediates in our synthetic investigations of antiviral drugs.  We report here its crystal structure. As shown in Fig. 1, the dihedral angle of 35.8 (6)° and 81.6 (6)° between the connecting sulfourea unit and the benzene ring, and between the 2-chloro-benzene ring and the connecting sulfourea group, respectively. A combination of intermolecular π-π packing interaction, N—H···O and N—H···S hydrogen bonds help stabilize the structure. In addition, weak C—H···π interactions are also present.

### Experimental

A solution of 0.23 g (3 mmol) of ammonium thiocyanate in 7 ml of acetonitrile was added to a solution of 0.52 g (3 mmol) of 2-chlorobenzoyl chloride in 2.5 ml of toluene. The mixture was heated for 5 min at 40°C and filtered from ammonium chloride, the filtrate was added to a solution of 0.32 g (3 mmol) of phenylmethanamine in 5 ml of acetonitrile, the mixture was stirred for 3 h at room temperature and evaporated, and the residue was washed with ethanol and recrystallized from ethanol. Yield 0.77 g (85%). Crystals suitable for X-ray analysis were obtained by slow evaporation from a solution of dichloromethane.

### Refinement

Amine hydrogens were located in a difference map and refined freely. The reminaing H atoms were positioned geometrically (C—H = 0.93–0.97 Å) and refined using a riding model, with *U*_iso_(H) = 1.2–1.5*U*_eq_(C).

### Crystal data

**Table:**

C_15_H_13_ClN_2_OS	*Z* = 2
*M**_r_* = 304.78	*F*(000) = 316
Triclinic, *P*1	*D*_x_ = 1.432 Mg m^−^^3^
Hall symbol: -P 1	Mo *K*α radiation, λ = 0.71073 Å
*a* = 7.347 (2) Å	Cell parameters from 2259 reflections
*b* = 9.658 (3) Å	θ = 3.2–27.5°
*c* = 11.003 (3) Å	µ = 0.41 mm^−^^1^
α = 110.150 (5)°	*T* = 153 K
β = 90.767 (3)°	Block, colorless
γ = 104.058 (3)°	0.40 × 0.30 × 0.30 mm
*V* = 707.0 (4) Å^3^	

### Data collection

**Table:**

Rigaku AFC10/Saturn724+ diffractometer	2481 independent reflections
Radiation source: Rotating Anode	2194 reflections with *I* > 2σ(*I*)
graphite	*R*_int_ = 0.017
Detector resolution: 28.6 pixels mm^-1^	θ_max_ = 25.3°, θ_min_ = 3.2°
φ and ω scans	*h* = −8→8
Absorption correction: multi-scan (*ABSCOR*; Higashi, 1995)	*k* = −11→11
*T*_min_ = 0.852, *T*_max_ = 0.886	*l* = −13→13
5691 measured reflections	

### Refinement

**Table:**

Refinement on *F*^2^	Primary atom site location: structure-invariant direct methods
Least-squares matrix: full	Secondary atom site location: difference Fourier map
*R*[*F*^2^ > 2σ(*F*^2^)] = 0.029	Hydrogen site location: inferred from neighbouring sites
*wR*(*F*^2^) = 0.073	H atoms treated by a mixture of independent and constrained refinement
*S* = 1.01	*w* = 1/[σ^2^(*F*_o_^2^) + (0.0368*P*)^2^ + 0.265*P*] where *P* = (*F*_o_^2^ + 2*F*_c_^2^)/3
2481 reflections	(Δ/σ)_max_ < 0.001
189 parameters	Δρ_max_ = 0.23 e Å^−^^3^
0 restraints	Δρ_min_ = −0.18 e Å^−^^3^

### Special details

**Table:**

Geometry. All e.s.d.'s (except the e.s.d. in the dihedral angle between two l.s. planes) are estimated using the full covariance matrix. The cell e.s.d.'s are taken into account individually in the estimation of e.s.d.'s in distances, angles and torsion angles; correlations between e.s.d.'s in cell parameters are only used when they are defined by crystal symmetry. An approximate (isotropic) treatment of cell e.s.d.'s is used for estimating e.s.d.'s involving l.s. planes.
Refinement. Refinement of *F*^2^ against ALL reflections. The weighted *R*-factor *wR* and goodness of fit *S* are based on *F*^2^, conventional *R*-factors *R* are based on *F*, with *F* set to zero for negative *F*^2^. The threshold expression of *F*^2^ > σ(*F*^2^) is used only for calculating *R*-factors(gt) *etc*. and is not relevant to the choice of reflections for refinement. *R*-factors based on *F*^2^ are statistically about twice as large as those based on *F*, and *R*- factors based on ALL data will be even larger.

### Fractional atomic coordinates and isotropic or equivalent isotropic displacement parameters (Å^2^)

**Table:**

	*x*	*y*	*z*	*U*_iso_*/*U*_eq_	
Cl1	0.69955 (6)	0.10088 (5)	0.08296 (4)	0.03365 (14)	
S1	0.81096 (6)	0.64421 (5)	0.00524 (4)	0.02590 (13)	
O1	0.71779 (16)	0.46704 (14)	0.34073 (11)	0.0283 (3)	
N1	0.84005 (19)	0.48399 (15)	0.15434 (13)	0.0203 (3)	
N2	0.62797 (19)	0.63073 (16)	0.20842 (13)	0.0219 (3)	
C1	0.9133 (2)	0.18430 (18)	0.18156 (14)	0.0202 (3)	
C2	1.0373 (2)	0.09656 (19)	0.18009 (15)	0.0243 (4)	
H2	1.0062	−0.0087	0.1267	0.029*	
C3	1.2074 (2)	0.1651 (2)	0.25780 (16)	0.0264 (4)	
H3	1.2953	0.1069	0.2562	0.032*	
C4	1.2512 (2)	0.3177 (2)	0.33806 (16)	0.0261 (4)	
H4	1.3678	0.3632	0.3919	0.031*	
C5	1.1247 (2)	0.40367 (18)	0.33975 (15)	0.0226 (3)	
H5	1.1539	0.5079	0.3956	0.027*	
C6	0.9550 (2)	0.33762 (17)	0.25978 (14)	0.0181 (3)	
C7	0.8239 (2)	0.43410 (17)	0.25728 (15)	0.0192 (3)	
C8	0.7526 (2)	0.58559 (17)	0.12955 (14)	0.0193 (3)	
C9	0.5298 (2)	0.74124 (19)	0.19805 (16)	0.0254 (4)	
H9A	0.4528	0.6995	0.1126	0.030*	
H9B	0.6234	0.8366	0.2031	0.030*	
C10	0.4041 (2)	0.77650 (17)	0.30540 (15)	0.0201 (3)	
C11	0.4601 (2)	0.79494 (18)	0.43245 (15)	0.0227 (3)	
H11	0.5805	0.7838	0.4530	0.027*	
C12	0.3419 (2)	0.82945 (18)	0.52955 (16)	0.0253 (4)	
H12	0.3805	0.8400	0.6158	0.030*	
C13	0.1679 (2)	0.84838 (18)	0.50053 (17)	0.0279 (4)	
H13	0.0868	0.8722	0.5668	0.033*	
C14	0.1120 (2)	0.83262 (19)	0.37480 (17)	0.0285 (4)	
H14	−0.0065	0.8474	0.3552	0.034*	
C15	0.2287 (2)	0.79521 (19)	0.27727 (16)	0.0248 (4)	
H15	0.1884	0.7823	0.1907	0.030*	
H1N	0.924 (3)	0.459 (2)	0.1044 (18)	0.030 (5)*	
H2N	0.605 (3)	0.593 (2)	0.2644 (19)	0.032 (5)*	

### Atomic displacement parameters (Å^2^)

**Table:**

	*U*^11^	*U*^22^	*U*^33^	*U*^12^	*U*^13^	*U*^23^
Cl1	0.0275 (2)	0.0300 (2)	0.0343 (3)	0.00316 (18)	−0.00924 (18)	0.00382 (19)
S1	0.0321 (2)	0.0347 (3)	0.0234 (2)	0.01932 (19)	0.01270 (17)	0.01826 (19)
O1	0.0315 (7)	0.0388 (7)	0.0274 (6)	0.0197 (6)	0.0153 (5)	0.0201 (6)
N1	0.0235 (7)	0.0238 (7)	0.0197 (7)	0.0131 (6)	0.0087 (6)	0.0103 (6)
N2	0.0253 (7)	0.0261 (7)	0.0240 (7)	0.0137 (6)	0.0106 (6)	0.0158 (6)
C1	0.0195 (8)	0.0234 (8)	0.0171 (8)	0.0041 (6)	0.0018 (6)	0.0077 (7)
C2	0.0319 (9)	0.0219 (8)	0.0223 (8)	0.0115 (7)	0.0074 (7)	0.0086 (7)
C3	0.0250 (9)	0.0334 (9)	0.0304 (9)	0.0158 (7)	0.0087 (7)	0.0176 (8)
C4	0.0183 (8)	0.0344 (9)	0.0298 (9)	0.0058 (7)	0.0004 (7)	0.0175 (8)
C5	0.0233 (8)	0.0213 (8)	0.0228 (8)	0.0030 (7)	0.0018 (6)	0.0095 (7)
C6	0.0194 (8)	0.0211 (8)	0.0171 (8)	0.0062 (6)	0.0062 (6)	0.0103 (7)
C7	0.0193 (8)	0.0198 (8)	0.0187 (8)	0.0041 (6)	0.0027 (6)	0.0080 (7)
C8	0.0200 (8)	0.0195 (8)	0.0189 (8)	0.0065 (6)	0.0025 (6)	0.0066 (7)
C9	0.0302 (9)	0.0292 (9)	0.0269 (9)	0.0174 (7)	0.0105 (7)	0.0157 (8)
C10	0.0223 (8)	0.0159 (7)	0.0248 (8)	0.0066 (6)	0.0066 (6)	0.0095 (7)
C11	0.0216 (8)	0.0228 (8)	0.0262 (9)	0.0071 (7)	0.0036 (6)	0.0108 (7)
C12	0.0308 (9)	0.0222 (8)	0.0226 (8)	0.0061 (7)	0.0059 (7)	0.0082 (7)
C13	0.0293 (9)	0.0228 (9)	0.0325 (10)	0.0089 (7)	0.0150 (7)	0.0094 (8)
C14	0.0207 (9)	0.0278 (9)	0.0393 (10)	0.0115 (7)	0.0066 (7)	0.0114 (8)
C15	0.0259 (9)	0.0256 (8)	0.0259 (9)	0.0109 (7)	0.0029 (7)	0.0101 (7)

### Geometric parameters (Å, °)

**Table:**

Cl1—C1	1.7409 (16)	C5—C6	1.390 (2)
S1—C8	1.6752 (16)	C5—H5	0.9500
O1—C7	1.2207 (19)	C6—C7	1.500 (2)
N1—C7	1.371 (2)	C9—C10	1.506 (2)
N1—C8	1.3913 (19)	C9—H9A	0.9900
N1—H1N	0.85 (2)	C9—H9B	0.9900
N2—C8	1.318 (2)	C10—C15	1.389 (2)
N2—C9	1.459 (2)	C10—C11	1.390 (2)
N2—H2N	0.82 (2)	C11—C12	1.388 (2)
C1—C2	1.383 (2)	C11—H11	0.9500
C1—C6	1.386 (2)	C12—C13	1.383 (2)
C2—C3	1.382 (2)	C12—H12	0.9500
C2—H2	0.9500	C13—C14	1.384 (3)
C3—C4	1.386 (2)	C13—H13	0.9500
C3—H3	0.9500	C14—C15	1.387 (2)
C4—C5	1.385 (2)	C14—H14	0.9500
C4—H4	0.9500	C15—H15	0.9500
			
C7—N1—C8	127.85 (13)	N2—C8—N1	116.48 (14)
C7—N1—H1N	116.5 (13)	N2—C8—S1	124.11 (12)
C8—N1—H1N	115.2 (13)	N1—C8—S1	119.41 (11)
C8—N2—C9	122.95 (14)	N2—C9—C10	110.88 (13)
C8—N2—H2N	117.3 (13)	N2—C9—H9A	109.5
C9—N2—H2N	119.8 (13)	C10—C9—H9A	109.5
C2—C1—C6	121.61 (15)	N2—C9—H9B	109.5
C2—C1—Cl1	119.44 (13)	C10—C9—H9B	109.5
C6—C1—Cl1	118.95 (12)	H9A—C9—H9B	108.1
C3—C2—C1	118.67 (15)	C15—C10—C11	118.98 (14)
C3—C2—H2	120.7	C15—C10—C9	118.94 (14)
C1—C2—H2	120.7	C11—C10—C9	122.06 (14)
C2—C3—C4	120.76 (15)	C12—C11—C10	120.64 (15)
C2—C3—H3	119.6	C12—C11—H11	119.7
C4—C3—H3	119.6	C10—C11—H11	119.7
C5—C4—C3	119.94 (15)	C13—C12—C11	119.88 (16)
C5—C4—H4	120.0	C13—C12—H12	120.1
C3—C4—H4	120.0	C11—C12—H12	120.1
C4—C5—C6	120.07 (15)	C12—C13—C14	119.91 (15)
C4—C5—H5	120.0	C12—C13—H13	120.0
C6—C5—H5	120.0	C14—C13—H13	120.0
C1—C6—C5	118.92 (14)	C13—C14—C15	120.18 (15)
C1—C6—C7	121.51 (14)	C13—C14—H14	119.9
C5—C6—C7	119.54 (14)	C15—C14—H14	119.9
O1—C7—N1	124.11 (14)	C14—C15—C10	120.39 (15)
O1—C7—C6	122.83 (13)	C14—C15—H15	119.8
N1—C7—C6	113.03 (13)	C10—C15—H15	119.8
			
C6—C1—C2—C3	−0.7 (2)	C5—C6—C7—N1	98.18 (17)
Cl1—C1—C2—C3	179.23 (12)	C9—N2—C8—N1	177.65 (14)
C1—C2—C3—C4	1.6 (2)	C9—N2—C8—S1	−1.7 (2)
C2—C3—C4—C5	−0.8 (2)	C7—N1—C8—N2	−5.7 (2)
C3—C4—C5—C6	−0.8 (2)	C7—N1—C8—S1	173.68 (13)
C2—C1—C6—C5	−0.9 (2)	C8—N2—C9—C10	−177.02 (15)
Cl1—C1—C6—C5	179.18 (11)	N2—C9—C10—C15	−141.11 (15)
C2—C1—C6—C7	177.52 (14)	N2—C9—C10—C11	40.6 (2)
Cl1—C1—C6—C7	−2.4 (2)	C15—C10—C11—C12	0.9 (2)
C4—C5—C6—C1	1.6 (2)	C9—C10—C11—C12	179.12 (15)
C4—C5—C6—C7	−176.77 (14)	C10—C11—C12—C13	−1.1 (2)
C8—N1—C7—O1	4.5 (3)	C11—C12—C13—C14	0.1 (2)
C8—N1—C7—C6	−173.81 (14)	C12—C13—C14—C15	1.1 (3)
C1—C6—C7—O1	101.52 (19)	C13—C14—C15—C10	−1.4 (3)
C5—C6—C7—O1	−80.1 (2)	C11—C10—C15—C14	0.4 (2)
C1—C6—C7—N1	−80.19 (18)	C9—C10—C15—C14	−177.92 (15)

### Hydrogen-bond geometry (Å, °)

**Table:**

*D*—H···*A*	*D*—H	H···*A*	*D*···*A*	*D*—H···*A*
N1—H1N···S1^i^	0.86 (2)	2.53 (2)	3.3698 (18)	166.2 (18)
N2—H2N···O1	0.82 (2)	2.01 (2)	2.669 (2)	137 (2)

Symmetry codes: (i) −*x*+2, −*y*+1, −*z*.
